# Caregiver Burden and Neuro‐Oncology Patient Function: Impacts on Relationship Quality

**DOI:** 10.1002/pon.70634

**Published:** 2026-10-18

**Authors:** Maija Reblin, Deanna Witte, Kristen J. Wells, Peter Forsyth, Margaret M. Byrne

**Affiliations:** ^1^ Department of Family Medicine University of Vermont Burlington Vermont USA; ^2^ Department of Health Outcomes & Behavior Moffitt Cancer Center Tampa Florida USA; ^3^ Department of Psychology San Diego State University San Diego California USA; ^4^ Neuro‐Oncology Program Moffitt Cancer Center Tampa Florida USA

**Keywords:** brain neoplasms, caregiver, caregiver burden, interpersonal relations, symptom burden

## Abstract

**Background:**

Prior work has identified the protective effects of relationship quality on cancer outcomes. However, less work has focused on how the cancer experience can change relationship quality.

**Aims:**

To describe neuro‐oncology patient‐caregiver relationship quality over time and identify the impact of caregiver burden and patient physical and cognitive function on perceptions of relationship quality.

**Methods:**

This is a secondary analysis of data obtained at enrollment and 8 weeks. Actor‐partner interdependence models of patient and caregiver ratings of their relationship to each other (Couples Satisfaction Index) over time also included baseline measures of patient cognitive and physical function (Neuro‐QOL), caregiver burden (Zarit), and change in these measures.

**Results:**

Data from 124 neuro‐oncology patient‐caregiver dyads were analyzed. Relationship quality at both time points, on average, indicated non‐distressed relationships, but patients rated relationship quality significantly better than caregivers. Both rated relationship quality significantly worse over time (*p* values < 0.022). At baseline, caregiver burden was significantly negatively associated with patient (*p* = 0.014) and caregiver relationship quality (*p* < 0.001). Patient and caregiver baseline relationship quality predicted their own 8‐week scores (actor effect; *p* values < 0.001). Baseline patient cognitive function (*p* = 0.011) and change in patient cognitive function (*p* = 0.039) were positively associated with 8‐week patient relationship quality. Change in caregiver burden was negatively associated with 8‐week caregiver relationship quality (*p* < 0.001).

**Conclusions:**

Prior research has largely focused on relationship quality as a resource, but factors associated with the cancer experience may impact perceptions of relationship quality. Lowering caregiver burden may be beneficial to preserving this important coping resource.

Brain tumors often progress rapidly, and there is poor prognosis associated with the most common types of primary tumors in adults. Both the cancer and its treatment can increase patient physical disability, and many patients also experience cognitive dysfunction and personality changes [1]. As a result, many patients with brain tumors rely on family members or friends to act as caregivers. While caregivers report that providing care can be meaningful [2], caring for a person with a brain tumor is also associated with high levels of burden [3]. Both patients with brain tumor and their caregivers often experience negative impacts to quality of life after diagnosis [3, 4]. Importantly, brain tumor treatment and management of sequelae occur within the context of the existing patient and caregiver relationship, most commonly a spousal or parent‐child relationship. A high‐quality relationship can serve as a source of strength and resource for social support or dyadic coping for both patients and caregivers managing a cancer experience, particularly one as serious as brain cancer [5, 6]. Having higher quality relationships has been associated with better quality of care [7] and lower perceptions of stress for both cancer patients and caregivers [6, 8].

As indicated above, most prior work has focused on the protective effects of relationship quality on health outcomes [9] or enhancing existing relationships to improve psychosocial interventions [10]. Many of these studies rely on baseline assessments of relationship quality. Yet, there is some evidence that relationship quality can change over time [11, 12]. Additionally, some data from older adults suggests that worse health is related to lower relationship satisfaction [13] and factors like physical and cognitive fatigue may be associated with poorer relationship quality in both cancer patients and their caregivers [14]. More caregiver burden has also been shown to lead to lower relationship satisfaction [15].

Mechanisms explaining this association have not been widely explored. However, it has been documented that cancer can make maintaining a personal relationship, including emotional exchange, less of a priority, in favor of a caregiver‐patient relationship, focused more on addressing one person's health needs [5, 16]. For example, in the context of cancer caregiving, patients or caregivers may limit important emotional exchange in the interest of trying to protect each other, but this can limit intimacy [17]. Existing work to explore how patient function and caregiver burden impact relationship quality across cancer types is often limited by cross‐sectional data collection or assessments of relationship quality only from one member of the dyad.

Further, the context of cancer and caregiving experience may be important. Many studies focus on longer‐term care situations, and when longitudinal data exists, often assessments span periods of time from 2 to 3 months to years [9, 12, 18], However, little work exists in contexts with more rapid, intense care needs, such as the context of brain cancer. The neurodegenerative nature of brain cancer is unique and may particularly impact perception of relationship quality in ways not seen in other cancers. Yet, the relatively rapid disease progression may make adjustment to change in patient function and caregiver burden more difficult than slower‐progressing cancers or dementia contexts [19]. Namely, the 5 year relative survival rate for glioblastoma, the most common primary malignant brain tumor, is 5%–10%, compared to 35% for advanced cancers overall and between 25% and 70% for various forms of dementia [20, 21]. This may make changes in relationship quality more apparent in a shorter time‐frame than seen in most existing studies.

Prior work with high grade glioma patients and their caregivers shows that symptom burden impacted patient perceptions of relationships and caregiver burden impacted caregiver perceptions of relationships. However, the sample size was small, limiting the ability to demonstrate more complex relationships among variables [22]. As such, there is a need to elucidate better how the relationship quality between patients with brain tumor and their caregivers changes over time. Our goal was to describe relationship quality between patients with brain tumor and their caregivers over 8 weeks and to identify the impact of caregiver burden and patient physical and cognitive function at baseline on patient and caregiver relationship quality at baseline and 8 weeks.

## 1 Method

### 1.1 Study Design

This is a secondary analysis of data collected as part of a supportive care trial for caregivers of patients with brain tumors [23]. In this study, baseline data were collected from patients and caregivers at enrollment and follow‐up data were collected at 8 weeks, the primary study endpoint. The intervention, which was directed only at caregivers, had no significant effect on variables included in the current analysis; as such, both intervention and control participants are included together and we do not control for condition. The parent study was registered with clinicaltrials.gov prior to participant enrollment (NCT04268979). All study procedures were approved by the Advarra Institutional Review Board (Pro00029204).

### 1.2 Participants

Patient and caregiver participants were recruited from the neuro‐oncology clinic of a large National Cancer Institute (NCI)‐designated Comprehensive Cancer Center in Florida from February 2020 to June 2024. All clinic patients were screened for potential eligibility, and those who were potentially eligible were approached in clinic, by letter, or by phone; interested patients were asked to nominate a family caregiver for participation. Caregivers were also approached in clinic or by phone.

Inclusion criteria for all participants were: (1) age 21+ years, (2) English‐speaking, and (3) able to complete questionnaires (including by proxy). Caregivers had to self‐identify as being a primary caregiver of a patient with a brain tumor. Patients were within 9 months of diagnosis of a new or recurrent brain tumor (primary or secondary), undergoing active treatment, with a prognosis of at least 6 months, and a Karnofsky Performance Scale score ≥ 50 (determined by oncologist). Patients and caregivers were required to enroll as pairs.

### 1.3 Procedures

All consented participants completed online or paper‐based baseline questionnaires, including demographics, caregiving characteristics, health information, and psychosocial measures, including those described below, upon enrollment. Psychosocial measures were repeated at 8 weeks.

### 1.4 Measures

Relationship quality was assessed at baseline and 8 weeks for patients and caregivers using the 4‐item adapted version of the Couple Satisfaction Index [24]. The 4‐item version items focuses on degree of happiness, comfort, reward in the relationship, as well as a general assessment of satisfaction. To account for non‐romantic relationships, the original wording of partner was changed to *patient/caregiver.* The CSI‐4 has been previously used in other caregiving populations, including non‐romantic pairs [25, 26]. Participants rated items on a 6‐point scale (“not at all” to “completely”). All items are summed, and a score of 13.5 or lower indicates notable relationship dissatisfaction [24].

Patient physical functioning was assessed using the 8‐item Neuro Quality of Life (Neuro QOL) Lower Extremity Function questionnaire. Patient cognitive functioning was assessed using the 8‐item Neuro Quality of Life (Neuro QOL) Cognitive Function questionnaire. Both measures were completed at baseline and 8 weeks. Neuro‐QOL measures have been developed to be psychometrically‐sound for individuals with neurological conditions [27]. While most patients completed their own questionnaires, caregiver proxy results are also valid [28]. Items are summed, and higher scores indicate better patient functioning [29]. In our analysis, we use raw baseline scores and change scores (8 weeks minus baseline; lower change score means decreased function). Raw scores can be converted to standardized scores (with *M* = 50, SD = 10) via the Neuro‐QOL scoring manual for interpretation [30].

Caregiver burden was assessed using the 12‐item Zarit Burden Interview Short Form [31] at baseline and 8 weeks. Caregivers rate items related to subjective burden on a 5‐point Likert scale (Ranging from “never” to “nearly always”), and item ratings are summed to calculate a total score. The total score ranges from 0 to 48 (0–10 no to mild burden; 10–20 mild to moderate burden; over 20 high burden). In our analysis, we use baseline scores and change scores (8 weeks minus baseline; a higher change score means increased burden).

### 1.5 Analysis

Descriptive analysis was conducted on all variables. Mplus software was used to compute an actor‐partner interdependence model (APIM) of patient and caregiver perceptions of relationship quality at study enrollment (predictors) and at 8 weeks (outcomes) using structural equation modeling. Because relationship quality is a dyadic‐level construct, reports from both members are important, yet are highly correlated. APIMs are used to account for interdependence between patient and caregiver reports of predictors and outcomes to estimate within‐ (actor) and between‐partner (partner) effects.

As shown in Figure 1, we also include multivariate predictors of relationship quality as covariates in the model: baseline measures of patient cognitive function, patient physical function, and caregiver burden, and change in each of these measures between enrollment and 8 weeks. Correlations between predictor variables were assessed. There were no significant differences in a priori analysis of included variables by intervention group, clinical, or demographic characteristics, so other covariates were not included in our model.

**FIGURE 1:**
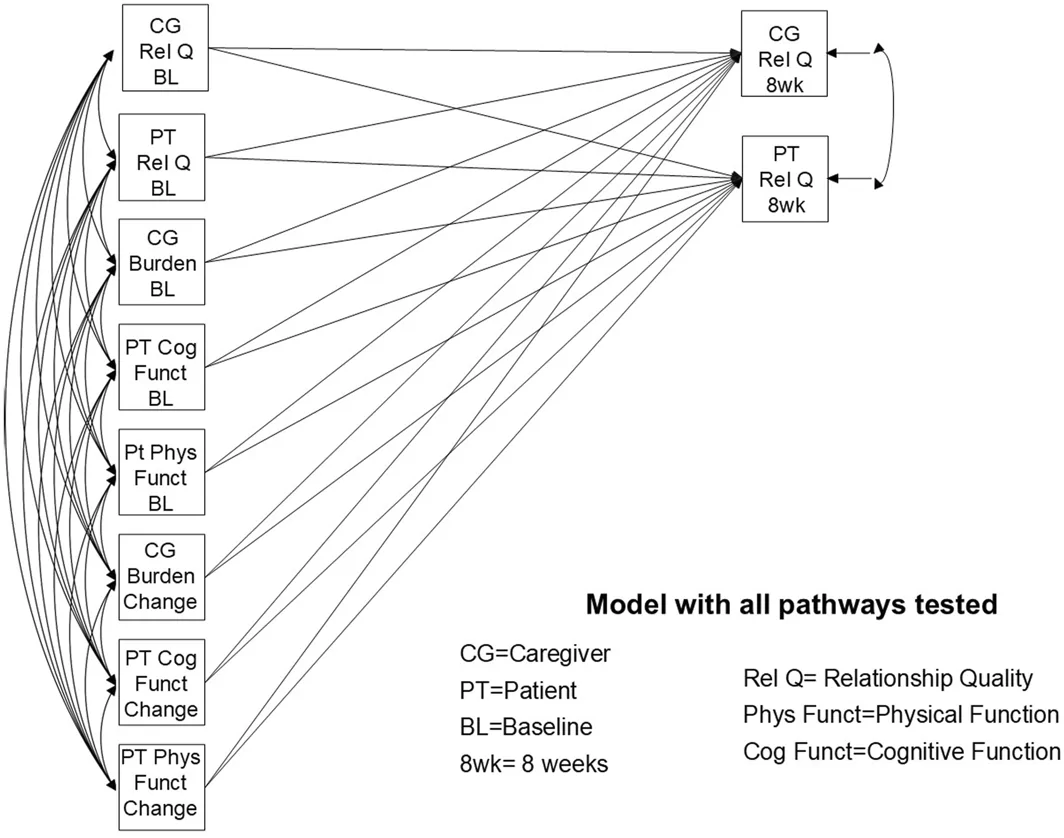
Model with all pathways tested. 8 wk = 8 weeks; BL = Baseline; CG = Caregiver; Cog Funct = Cognitive Function; Phys Funct = Physical Function; PT = Patient; Rel *Q* = Relationship Quality.

Maximum likelihood estimation was used to estimate population parameters that included missing data from all observed data, and the data were bootstrapped (*n* = 2000) to correct for potentially biased distributions. Four indices were used to evaluate the model fit: nonsignificant chi‐squared values (*p* > 0.05), the comparative fit index > 0.95, the root mean squared error of approximation < 0.06, and the standardized root mean squared residual < 0.08, which indicate adequate fit of a specified model to the data [32]. Although 200+ dyads are required to detect small partner effects, only 50–80 are required to detect medium actor effects [33]. In this analysis, we balance statistical rigor with the difficulty in recruiting dyadic data in specialized populations [33], including neuro‐oncology patient‐caregiver dyads.

## 2 Results

### 2.1 Descriptive Analysis

Data from 124 brain cancer patient‐caregiver dyads were analyzed. Demographic data are presented in Table 1. The majority of caregivers were female (66%), White (88%), and the spouse of the patient (77%), with an average age of 57.5 years (SD = 14.7 years). The average length of the patient‐caregiver relationship was 28.6 years (SD = 15.4). Patients most commonly were diagnosed with glioblastoma (63%), 58% were male, 90% were White, with an average age of 57.5 years (SD = 14.9 years). The median time from diagnosis to study consent was 97 days.

**TABLE 1:** Demographic, health, and psychosocial information at baseline.

		Caregivers (*n* = 124)	Patients (*n* = 124)
Variable	Levels	*M* (SD)	*M* (SD)
Age (years)		57.5 (14.7)	57.5 (14.9)
Length of patient‐caregiver relationship (years)		28.6 (15.4)	

		***N* (%)**	***N* (%)**
Gender	Male	35 (28%)	71 (58%)
	Female	82 (66%)	50 (40%)
	Missing	7 (6%)	16 (12%)
Race (all that apply)	American Indian or Alaskan Native	2 (2%)	0 (0%)
	Asian	0 (0%)	3 (3%)
	Black or African American	4 (3%)	7 (6%)
	Middle Eastern or North African	1 (1%)	0 (0%)
	Native Hawaiian or other Pacific Islander	0 (0%)	1 (1%)
	White or Caucasian	109 (88%)	110 (90%)
	Some other race, ethnicity, or origin	4 (3%)	2 (2%)
Ethnicity	Non‐Hispanic	109 (88%)	116 (93%)
	Hispanic	7 (6%)	5 (3%)
	Missing/prefer not to answer	8 (6%)	3 (3%)
Education	Less than high school diploma/equivalent	1 (1%)	2 (2%)
	High school diploma or equivalent	16 (13%)	13 (10%)
	Some college	37 (30%)	48 (38%)
	Four‐year college degree	32 (26%)	30 (24%)
	Postgraduate degree	31 (25%)	26 (21%)
	Other	0 (0%)	1 (1%)
	Missing/unknown	7 (6%)	4 (3%)
Work hours per week	≥ 35 hours	39 (32%)	28 (22%)
	< 35 hours	6 (5%)	2 (2%)
	Not working for pay	70 (57%)	87 (71%)
	Prefer not to answer/missing	9 (7%)	7 (6%)
Financial situation	Not very good	20 (16%)	25 (17%)
	Comfortable	67 (54%)	58 (47%)
	More than adequate to meet my needs	25 (20%)	32 (26%)
	Prefer not to answer/missing	12 (10%)	9 (7%)
Relationship to patient	Spouse/partner	95 (77%)	
	Parent/in‐law	7 (6%)	
	Sibling/in‐law	2 (1%)	
	Child	9 (7%)	
	Friend/neighbor	2 (1%)	
	Other	2 (1%)	
	Missing	7 (6%)	
Length of care for patient	1–3 months	16 (22%)	
	4–6 months	25 (34%)	
	7–12 months	12 (16%)	
	1–2 years	9 (12%)	
	3–5 years	7 (10%)	
	More than 5 years	4 (5%)	
Patient diagnosis	Glioblastoma		78 (63%)
	Astrocytoma		13 (10%)
	Oligodendroglioma		4 (3%)
	Glioma (not otherwise specified)		5 (4%)
	Meningioma		7 (5%)
	Medulloblastoma		1 (1%)
	Brain metastasis		14 (11%)
	Leptomeningeal disease		2 (2%)
Primary brain tumor	No		16 (13%)
	Yes		108 (87%)
Patient WHO grade	1		2 (2%)
	2		6 (5%)
	3		12 (10%)
	4		85 (68%)
	Not in medical record/missing		19 (15%)

Descriptive data on key analytic constructs are shown in Table 2. Relationship quality was good for both patients and caregivers on average at both time points, with means above the distressed cut‐off. Patient ratings of the relationship were significantly higher than caregiver ratings at both baseline (*t* = 3.427, df = 73, *p* = 0.001) and 8 weeks (*t* = 2.369, df = 48, *p* = 0.022). Relationship quality ratings significantly declined over time for both patients (*t* = 2.442, df = 52, *p* = 0.018) and caregivers (*t* = 2.399, df = 90, *p* = 0.018).

**TABLE 2:** Means and standard deviations of caregiver and patient key constructs.

	Caregiver		Patient	
Construct	Baseline	8 week	Baseline	8 week
Relationship quality	16.02 (4.86)	15.56 (4.66)	17.47 (3.72)	16.73 (3.76)
Burden	13.59 (9.54)	13.77 (9.15)		
Cognitive function			25.91 (9.46)	25.68 (9.37)
Physical function			32.38 (7.89)	32.08 (8.68)

Caregiver burden on average was mild‐to‐moderate at baseline and increased slightly on average over time (Mchange = 0.76, SD = 6.55). Patient physical and cognitive function at baseline on average was about 1 SD below the benchmarked average (Physical *t* score = 40.8; cognitive *t* score = 40.9) and both decreased slightly on average over time.

### 2.2 Actor‐Partner Interdependence Model

The APIM had excellent fit characteristics, with all criteria met. Interdependence in patient and caregiver ratings of relationship quality at baseline was met (*p* = 0.004). Significant pathways in the model are shown in Figure 2.

**FIGURE 2:**
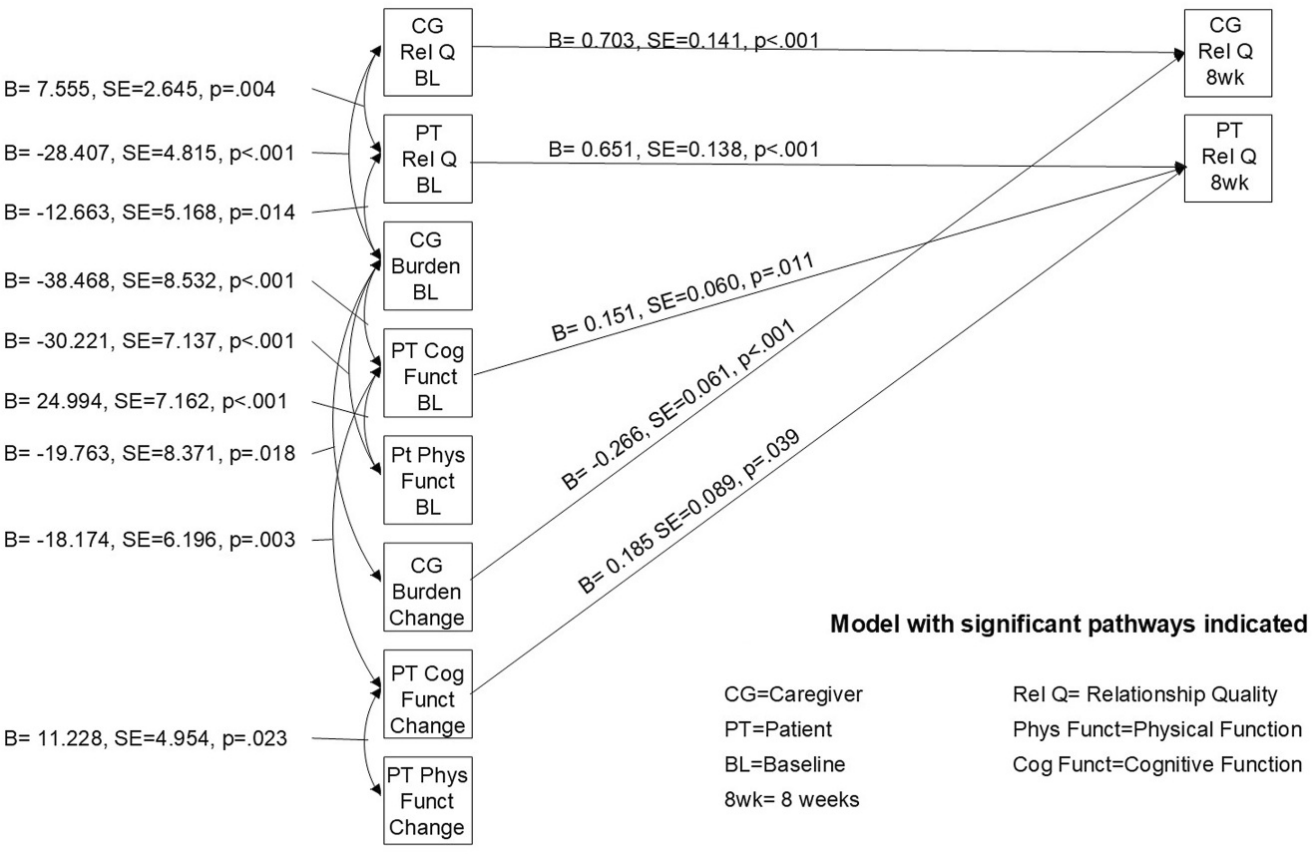
Model with significant pathways indicated. 8 wk = 8 weeks; BL = Baseline; CG = Caregiver; Cog Funct = Cognitive Function; Phys Funct = Physical Function; PT = Patient; Rel *Q* = Relationship Quality.

At baseline, caregiver burden was significantly negatively associated with both patient (*B* = −12.663, SE = 5.168, *p* = 0.014) and caregiver perception of relationship quality (*B* = −28.407, SE = 4.185, *p* < 0.001), but there was no significant association with change in burden or patient cognitive or physical function variables to relationship quality variables at baseline. There were significant relationships between baseline caregiver burden, patient cognitive and physical function (*p*s < 0.001) such that patient cognitive and physical function were positively related to each other and both were negatively related to caregiver burden. Change in patient cognitive function was positively related to change in patient physical function (*p* = 0.018), likely reflecting overall patient decline. More change in patient cognitive function was negatively associated with baseline cognitive function (*p* = 0.003), such that greater change was more likely when cognitive function started lower. More change in caregiver burden was negatively related to baseline caregiver burden (*p* = 0.018); again, greater change was more likely when burden started lower.

Patient perception of relationship quality at 8 weeks was significantly predicted by patient perception of relationship quality at baseline (actor effect; *B* = 0.651, SE = 1.838, *p* < 0.001), but there was no partner effect. Patient relationship quality at 8 weeks was also predicted by baseline patient cognitive function (*B* = 0.151, SE = 0.606, *p* = 0.011) and change in patient cognitive function (*B* = 0.185, SE = 0.089, *p* = 0.039), such that better cognitive function at baseline and more change in cognitive function (often decline) were associated with better patient perception of relationship quality at 8 weeks.

Caregiver perception of relationship quality at 8 weeks was significantly predicted by caregiver perception of relationship quality at baseline (actor effect; *B* = 0.703, SE = 0.141, *p* < 0.001), but there was no partner effect. Caregiver relationship quality was also predicted by change in burden (*B* = −0.266, SE = 0.061, *p* < 0.001) such that less change in burden over time (often less increase) was associated with better caregiver perception of relationship quality at 8 weeks.

## 3 Discussion

This study is one of the first to describe the change in relationship quality over time in patients with brain tumor and their caregivers. Prior research has largely focused on relationship quality as a resource. However, we focus on how the cancer experience, namely patient function and caregiver burden, may impact perceptions of relationship quality. In our data, relationship quality was generally good on average at both time points, and values were similar to other data collected from breast cancer patients about their partners [8]. Also in line with prior work, patients had statistically significantly better perceptions of the relationship than caregivers at each assessment [6], and both patient and caregiver perceptions of the relationship quality statistically significantly declined over time [11]. Though, on average, all dyad members were satisfied with their relationships, patients may see more mutuality and collaboration [34] as dyads cope together to manage the cancer, while caregivers may feel less reciprocity as they take over more tasks and manage the unique stressor of caregiving with less support from the patient [6].

Unsurprisingly, there were cross‐sectional associative relationships between patient function and caregiver burden at baseline, confirming that caregiver's perceived burden is related to objective patient status. However, perceptions of relationship quality at baseline for both patients and caregivers were associated only with baseline caregiver burden scores, not with baseline patient function scores. This suggests that, at a single time point, caregiver perception of burden may be more important than objective patient status for perceptions of relationship quality for both dyad members. Caregiver burden has been shown to be a multi‐faceted and dynamic construct, but prior research indicates that subjective burden, as measured by the Zarit Burden Scale, often remains stable as objective burden increases [19]. This may indicate that subjective burden reflects a perceived degree of difficulty, or inversely, level of adjustment. As caregivers adapt to or cope with their responsibilities, those responsibilities may thus seem less burdensome. The cross‐sectional relationship between caregiver burden and patient perception of relationship quality may also be an indicator that reflects this adaptation. Patients who perceive that they are a burden on the caregiver may feel less closeness or relationship satisfaction compared to patients who perceive that their caregivers are able to manage their care more effectively.

Longitudinally, the predictors of relationship quality at 8 weeks are intra‐ rather than interpersonal for both patients and caregivers. Patients' perception of relationship quality at 8 weeks was predicted by their own baseline cognitive function and change in cognitive function in addition to their own prior ratings of relationship quality. Prior work has shown that even mild cognitive deficits can detrimentally affect the ability to maintain relationships and engage effectively in social and occupational roles [35, 36]. Patients may have fewer cognitive resources to engage or feel unable to reciprocate if others take on larger roles, leading to social withdrawal or feelings of imbalance. However, most work has largely been cross‐sectional or focused on the protective effects of relationships on cognition [37]. In contrast, caregivers' perception of relationship quality at 8 weeks was predicted by their own change in perception of burden in addition to their prior ratings of the relationship. Again, this may be an indicator of adjustment, such that increased burden may indicate that caregivers may feel less confident or competent in their care. This feeling of competence may be independent of actual patient needs.

### 3.1 Limitations

Our sample had little variability in the types of relationships, such that caregivers were mostly female spouses, and most relationships were high quality. A larger, more diverse sample may allow for more power to identify smaller effects, especially for partner effects, and additional comparisons. For example, research suggests that gender differences in relationship quality variability arise more in lower‐satisfaction couples [38], and relationship satisfaction changes across the lifespan [39]. Additionally, the patient‐caregiver relationship does not exist in a vacuum and other factors not included in our analysis, such as the size of social networks, the quality of other family support, or communication styles, may be important in predicting changes in patient‐caregiver relationship quality [40]. Although all caregivers in our study identified as “primary” caregiver, variation exists in the amount of care contributed by others, which was not captured in this analysis. Our analysis also only focuses on a brief period during cancer treatment. In comparison to long‐term relationships, 8 weeks is a relatively short amount of time to see change, yet some signal does appear in our data. Additionally, the strongest predictor of later relationship quality ratings was one's own prior relationship quality rating. This suggests the importance of strong relationships prior to a cancer diagnosis and caregiving relationship and may point to additional upstream targets for future intervention. Future research could explore the development and evolution of relationships across the care trajectory, from before diagnosis through survivorship or end of life.

### 3.2 Clinical Implications

Our analysis suggests the importance of supporting family caregivers and reducing caregiver burden to protect caregiver and patient perception of relationship quality, a key resource for coping. Neuro‐oncology clinics should consider augmenting social work services to enhance the ability to offer tailored, systematic caregiver resources, including information and training, as well as referrals to additional programs, such as respite care, counseling, or financial assistance. Well‐supported caregivers are more likely to perceive lower levels of burden, which is associated with better quality relationship to the patient in our work, as well as better quality of care in the broader research literature [7].

### 3.3 Conclusion

In addition to high‐quality relationships being a resource for coping with cancer, we see that the experience of cancer can impact the perception of relationship quality for patients with brain tumors and their caregivers. While it may be challenging to protect cognitive function for these patients, improving perceptions of caregiver burden may protect caregiver and patient perception of their relationships, ultimately preserving this important resource for coping through the cancer trajectory.

## Author Contributions

M.R., M.M.B., K.W. contributed to study conception and design. Data preparation and review was performed by M.R., D.W. Data analysis was performed by M.R. The first draft of the manuscript was written by M.R. All authors commented on previous versions of the final manuscript. All authors read and approved the final manuscript.

## Funding

This work was supported by the National Cancer Institute, under award R01CA236034.

## Ethics Statement

This study was performed in line with the principles of the Declaration of Helsinki. Approval was granted by the Advarra Institutional Review Board (Pro00029204) prior to study implementation. Informed consent was obtained from all individual participants included in the study.

## Conflicts of Interest

The authors declare no conflicts of interest.

## Supporting information


Supporting Information S1

## Data Availability

The data that support the findings of this study are available on request from the corresponding author. The data are not publicly available due to privacy or ethical restrictions.
