# Supplementary material for: Caregiver Burden and Neuro‐Oncology Patient Function: Impacts on Relationship Quality
Source: Psychooncology. 2026 Oct 18;35(10):e70634. doi: 10.1002/pon.70634 (PMC13677109; doi:10.1002/pon.70634)
Supplement: Supplementary file 1 [file PON-35-e70634-s001.docx]

Supplement 1. Model results

|  | Unstandardized Co-efficient | | Standard Error | | Z score | P Value | Standardized Co-efficient |
| --- | --- | --- | --- | --- | --- | --- | --- |
| Outcome: CG Relationship Quality 8 Weeks | | | | | | | |
| CG Burden BL | -0.102 | | 0.065 | | -1.576 | 0.115 | -0.198 |
| PT Cognitive Function BL | 0.011 | | 0.054 | | 0.210 | 0.833 | 0.022 |
| PT Physical Function BL | 0.106 | | 0.076 | | 1.395 | 0.163 | 0.169 |
| CG Burden Change | -0.266 | | 0.061 | | -4.390 | 0.000 | -0.355 |
| PT Cognitive Function Change | 0.023 | | 0.086 | | 0.265 | 0.791 | 0.030 |
| PT Physical Function Change | -0.123 | | 0.142 | | -0.865 | 0.387 | -0.146 |
| PT Relationship Quality BL | -0.127 | | 0.195 | | -0.650 | 0.516 | -0.096 |
| CG Relationship Quality BL | 0.703 | | 0.141 | | 4.987 | 0.000 | 0.699 |
|  |  | |  | |  |  |  |
| Outcome: PT Relationship Quality 8 Weeks | | | | | | | |
| CG Burden BL | -0.074 | | 0.105 | | -0.707 | 0.480 | -0.185 |
| PT Cognitive Function BL | 0.151 | | 0.060 | | 2.536 | 0.011 | 0.376 |
| PT Physical Function BL | -0.135 | | 0.104 | | -1.295 | 0.195 | -0.280 |
| CG Burden Change | -0.004 | | 0.087 | | -0.047 | 0.963 | -0.007 |
| PT Cognitive Function Change | 0.185 | | 0.089 | | 2.069 | 0.039 | 0.315 |
| PT Physical Function Change | -0.165 | | 0.141 | | -1.170 | 0.242 | -0.253 |
| PT Relationship Quality BL | 0.651 | | 0.138 | | 4.716 | 0.000 | 0.636 |
| CG Relationship Quality BL | -0.033 | | 0.141 | | -0.232 | 0.817 | -0.042 |
|  | |  | |  |  |  |  |
| Outcome: CG Burden BL | | | | | | | |
| CG Relationship Quality BL | | -28.407 | | 4.815 | -5.899 | 0.000 | -0.185 |
| PT Relationship Quality BL | | -12.663 | | 5.168 | -2.450 | 0.014 | 0.376 |
|  | |  | |  |  |  |  |
| Outcome: PT Cognitive Function BL | | | | | | | |
| CG Relationship Quality BL | | 5.823 | | 4.800 | 1.213 | 0.225 | 0.127 |
| PT Relationship Quality BL | | 3.487 | | 4.116 | 0.847 | 0.397 | 0.100 |
| CG Burden BL | | -38.468 | | 8.532 | -4.508 | 0.000 | -0.430 |
|  | |  | |  |  |  |  |
| Outcome: PT Physical Function BL | | | | | | | |
| CG Relationship Quality BL | | 5.102 | | 3.392 | 1.504 | 0.133 | 0.134 |
| PT Relationship Quality BL | | 6.657 | | 3.927 | 1.695 | 0.090 | 0.230 |
| CG Burden BL | | -30.221 | | 7.137 | -4.235 | 0.000 | -0.406 |
| PT Cognitive Function BL | | 24.994 | | 7.162 | 3.490 | 0.000 | 0.338 |
|  | |  | |  |  |  |  |
| Outcome: CG Burden Change | | | | | | | |
| CG Relationship Quality BL | | 7.347 | | 3.946 | 1.862 | 0.063 | 0.232 |
| PT Relationship Quality BL | | 3.317 | | 4.892 | 0.678 | 0.498 | 0.138 |
| CG Burden BL | | -19.763 | | 8.371 | -2.361 | 0.018 | -0.320 |
| PT Cognitive Function BL | | 11.181 | | 7.935 | 1.409 | 0.159 | 0.182 |
| PT Physical Function BL | | -1.440 | | 7.023 | -0.205 | 0.838 | -0.028 |
|  | |  | |  |  |  |  |
| Outcome: PT Cognitive Function Change | | | | | | | |
| CG Relationship Quality BL | | 0.465 | | 3.572 | 0.130 | 0.896 | 0.015 |
| PT Relationship Quality BL | | 0.333 | | 3.329 | 0.100 | 0.920 | 0.014 |
| CG Burden BL | | -1.937 | | 6.624 | -0.292 | 0.770 | -0.032 |
| PT Cognitive Function BL | | -18.174 | | 6.196 | -2.933 | 0.003 | -0.299 |
| PT Physical Function BL | | 3.331 | | 6.877 | 0.484 | 0.628 | 0.066 |
| CG Burden Change | | -8.577 | | 6.886 | -1.246 | 0.213 | -0.204 |
|  | |  | |  |  |  |  |
| Outcome: PT Physical Function Change | | | | | | | |
| CG Relationship Quality BL | | -4.566 | | 3.017 | -1.513 | 0.130 | -0.161 |
| PT Relationship Quality BL | | 2.451 | | 3.242 | 0.756 | 0.450 | 0.114 |
| CG Burden BL | | -8.032 | | 6.297 | -1.275 | 0.202 | -0.146 |
| PT Cognitive Function BL | | 12.733 | | 7.352 | 1.732 | 0.083 | 0.232 |
| PT Physical Function BL | | -2.614 | | 5.679 | -0.460 | 0.645 | -0.057 |
| CG Burden Change | | -3.193 | | 7.079 | -0.451 | 0.652 | -0.084 |
| PT Cognitive Function Change | | 11.228 | | 4.954 | 2.266 | 0.023 | 0.300 |
|  | |  | |  |  |  |  |
| Outcome: CG Relationship Quality BL | | | | | | | |
| PT Relationship Quality BL | | 7.555 | | 2.645 | 2.856 | 0.004 | 0.420 |
|  | |  | |  |  |  |  |
| Outcome: PT Relationship Quality 8 weeks | | | | | | | |
| CG Relationship Quality 8 weeks | | 2.507 | | 1.283 | 1.954 | 0.051 | 0.407 |
